# Starvation Versus Developement

**Published:** 1884-06

**Authors:** 


### STARVATION VERSUS DEVELOPMENT.

SELF-DENIAL is a negative virtue; or rather it is the vice that poverty inflicts upon the unfortunate. A horse will digest all the food that can possibly be stuffed into him, provided he be properly exercised. The constant, strenuous cultivation of all a man's faculties will of itself preclude the cropping out of any enormities in one single direction. The truism that "all men are created free and equal" is not so absurd as our English cousins profess to believe it is. It is the neglect of a few faculties rather than over-indulgence of others that causes disease, misfortune and crime. As in social life the moment a man commences to go down hill everybody seems to be ready to give him a kick, so when a single faculty is neglected all the others seem ready to crowd it out of existence, though this is not really the case. The best man by wilful neglect of himself could bring himself to commit a murder, while the worst man by constant cultivation could be made to perform a generous, exalted deed. There is no crowding, no slaughtering among the faculties, but when one commences to decay the others apparently push ahead and exhibit themselves more prominently.

Instead, therefore, of forcing a boy to get up hungry from a dinner table, it is better to let him eat all his appetite requires, and then set him to work to convert that food into sound muscle. If that muscle is likely to make him too boisterous, he can be sent to school and church. If he shows signs of being doleful under that regimen, give him some amusement. It is not possible to transform a giant into a dwarf without first killing him, but it is very possible by full cultivation to make a dwarf grow into a giant. We are born with a full supply of faculties. An equal cultivation of all of them will ensure happiness. If a man has a passion for walking he should be allowed to walk to his heart's content, but he should be made to perform also some other exercise by which his arms, chest, body and neck could have ample opportunity for development. In the course of time he would not *want* to walk so much. It is not easy to go thoroughly into the details of this theory while limiting an article to a short space, but enough of it and there will be gathered from the statement that making a boy go hungry because he has a good appetite distresses his whole organization without benefitting any one faculty.

